# Investigation of natural and artificial radioactivity levels in travertines of the Cappadocia region in Turkey

**DOI:** 10.1007/s10653-024-01963-y

**Published:** 2024-05-02

**Authors:** M. Kamislioglu, I. Kocak, B. Buyuk, C. Eke, R. Ozaydin Ozkara, U. Temiz

**Affiliations:** 1 Department of Medical Imaging, Vocational School of Health Services, Bandirma Onyedi Eylul University, 10200 Balikesir, Turkey; 2 Boron Technologies Application and Research Center, Bandirma Onyedi Eylul University, 10200 Balikesir, Turkey; 3 Department of Engineering Science, Faculty of Engineering and Natural Sciences, Bandirma Onyedi Eylul University, 10200 Balikesir, Turkey; 4 Department of Mathematics and Science Education, Faculty of Education, Akdeniz University, 07058 Antalya, Turkey; 5 Nuclear Technology and Radiation Safety, Vocational School of Technical Sciences, Akdeniz University, 07058 Antalya, Turkey; 6 Geological Engineering Department, Engineering and Architectural Faculty, Yozgat Bozok University, 66100 Yozgat, Turkey

**Keywords:** Natural radionuclides, HpGe detector, Radiological dose parameters, Travertines, Radiation detection and measurement

## Abstract

This study determined natural and artificial radionuclide concentrations to evaluate natural radioactivity and health risk levels of nine travertines in the Yaprakhisar and Balkayası regions in Turkey. The samples coded B1-M, B2, B5, B7, B8, and B10 represent waste derived from the Yaprakhisar travertines, as well as samples T5-M, T12, and Z1 travertines derived from Balkayası. The levels of natural and artificial radionuclide concentrations (^232^Th, ^40^K, and ^137^Cs) were measured using a high-purity germanium (HpGe) detector system. The travertine activity ranged from 2.09 to 12.07 Bq kg^−1^ for ^232^Th, 4.21 to 13.41 Bq kg^−1^ for ^40^K, and 0.42–3.26 Bq kg^−1^ for ^137^Cs. The results showed that the activity concentration values for ^232^Th, ^40^K, and ^137^Cs were coherent with the travertine analysis results in the UNSCEAR, 2000; 2008 publications. The values obtained were lower than the average values in the UNSEAR reports. The radiological hazard parameters calculated in this study were absorbed gamma dose rate (D), radium equivalent activity (Ra_eq_), annual gonadal dose equivalent (AGDE), exposure dose (ER), total annual effective dose (AEDE_total_), excess lifetime cancer risk (ELCR_total_), gamma representative level (GRL), internal hazard index (H_in_) and external hazard index (H_ex_).

## Introduction

While investigating the impact of natural radioactivity on terrestrial environments, assessing the contribution of decay products from 238U and 232Th is crucial. By considering the decay rates of these radioactive isotopes, we can better understand their influence on the terrestrial level. Considering the Chernobyl and Japan nuclear power plant disasters, it has become essential to determine the affected areas and assess the impact of artificially emitted radioisotopes on ecosystems. Over the past 2 decades, there has been a growing interest in studying the levels of natural radioactivity in soil and rocks, aiming to uncover the damages caused and the extent of the effects of this anthropogenic ionizing radiation.

Investigators have conducted investigations on the levels of natural radioactivity in soil and rock samples from various regions in Turkey (Alshahri & El-Taher, 2019; Öztürk et al., 2013; Sahin et al., 2017; Shahbazi-Gahrouei et al., 2013; Yildirim et al., 2021).

Due to the location of Turkey within a tectonically active zone, limestone and marble are prevalent among the lithological formations. Furthermore, these areas' abundance of travertine formations can be attributed to favorable climatic conditions and geological factors. Travertines are sedimentary rocks formed due to geological, geomorphological, hydrographic, climatological, and biological processes (Baba & Sözbilir, 2012; Calvo & Regueiro, 2010). Travertine formations result from the evaporation of water containing calcium bicarbonate and are sedimentary deposits found in both hot and cold waters containing CaCO_3_. Travertines are formed and stored in various shapes depending on the geomorphological, climatological (pressure, temperature, evaporation, etc.), physical characteristics of groundwater (flow rate, dispersion, sedimentation, discharge), chemical, and biological properties. Turkey's abundant travertine deposits are utilized across multiple industries, from manufacturing lime and cement to creating unique souvenirs. These deposits take on different shapes like terraces, canals, cones, chimneys, or bridges, depending on the geological, geomorphological, hydrographic, and biological characteristics of the area where the water emerges. These hydrogeomorphological structures, essentially accumulations of travertines, are uncommon natural phenomena. Notable examples of these natural wonders can be observed in various locations across Turkey (Temiz et al., 2018, 2021). This study assesses the natural radiological risk of Yaprakhisar and Balkayasi travertines.

The study's focus, travertines, are terrestrial deposits that can be found on Earth in a variety of sizes and shapes. They are connected to hot geothermal resources rich in calcium and bicarbonate in active or recently active tectonic, volcanic, and geothermal regions and have a minimal distribution (De Filippis et al., 2012; Ford & Pedley, 1996; Temiz et al., 2018, 2021). Numerous geological, biological, archeological, and paleo-climatic studies use these carbonate formations.

In addition, travertines are used as building materials in the construction industry, like other natural rocks. For human health, it is crucial to understand the level of radioactivity in construction materials. Numerous recent investigations have been conducted on the natural radioactivity of building materials, including travertine (Alnour et al., 2012; Al-Zahrani, 2017; Del Claro et al., 2017; Dentoni et al., 2020; Ghiassi-Nejad et al., 2002; Kayakökü et al., 2016; Kumara et al., 2018; Marchetti et al., 2004; Nakhzari Moghadam, 2021; Smetsers & Tomas, 2019; Trevisi et al., 2005; Turhan et al., 2018; Yigitoglu et al., 2018).

Central Anatolian Volcanic Province (CAVP)'s Yaprakhisar and Balkayası travertines (Toprak & Göncüoǧlu, 1993) were examined in this study. The significance of the study is made clear by the fact that these travertines are processed and used as building materials. The results will form the basis for future studies to create the current radionuclide distribution map for the studied region (Yaprakhisar and Balkayası).

Various studies have been conducted in the literature to assess the radioactivity levels in neighboring regions such as the Mediterranean Sea, Cyprus, and Iran. These studies focused on quantifying the concentrations of natural and artificial radioactivity, explicitly emphasizing the isotopes ^226^Ra, ^232^Th, ^40^K, and ^137^Cs, particularly in soil and water samples (Abbasi, 2013; Abbasi et al., 2020a, 2020b; Abbasi, 2022; Abbasi et al., 2022a, 2022b, 2022c; Abbasi et al., A2022a, 2022b, 2022c; Abbasi & Mirekhtiary, 2013, 2019; Abbasi et al., 2020a, 2020b; Abbasi et al., 2022a, 2022b, 2022c; Kefalati et al., 2021).

The radioactivity concentrations of the samples were assessed by analyzing the gamma-ray spectra using a high-purity germanium detector (HPGe). These measurement outcomes were then used to compute various radiological parameters, including the absorbed gamma dose rate (D), radium equivalent activity (Ra_eq_), excess lifetime cancer risk (ELCR), annual effective dose equivalent (AEDE), exposure dose (ER), internal hazard index (H_in_), external hazard index (H_ex_), annual gonadal dose equivalent (AGDE), and Gamma index (Iγ) based on the radioactivity concentration values of the samples. These calculated results were subsequently compared to existing literature data and global average values. The findings of this study contribute to establishing a comprehensive database regarding natural radioactivity levels within the investigated area.

## Materials and methods

### Geology of study area

**Fig. 1:**
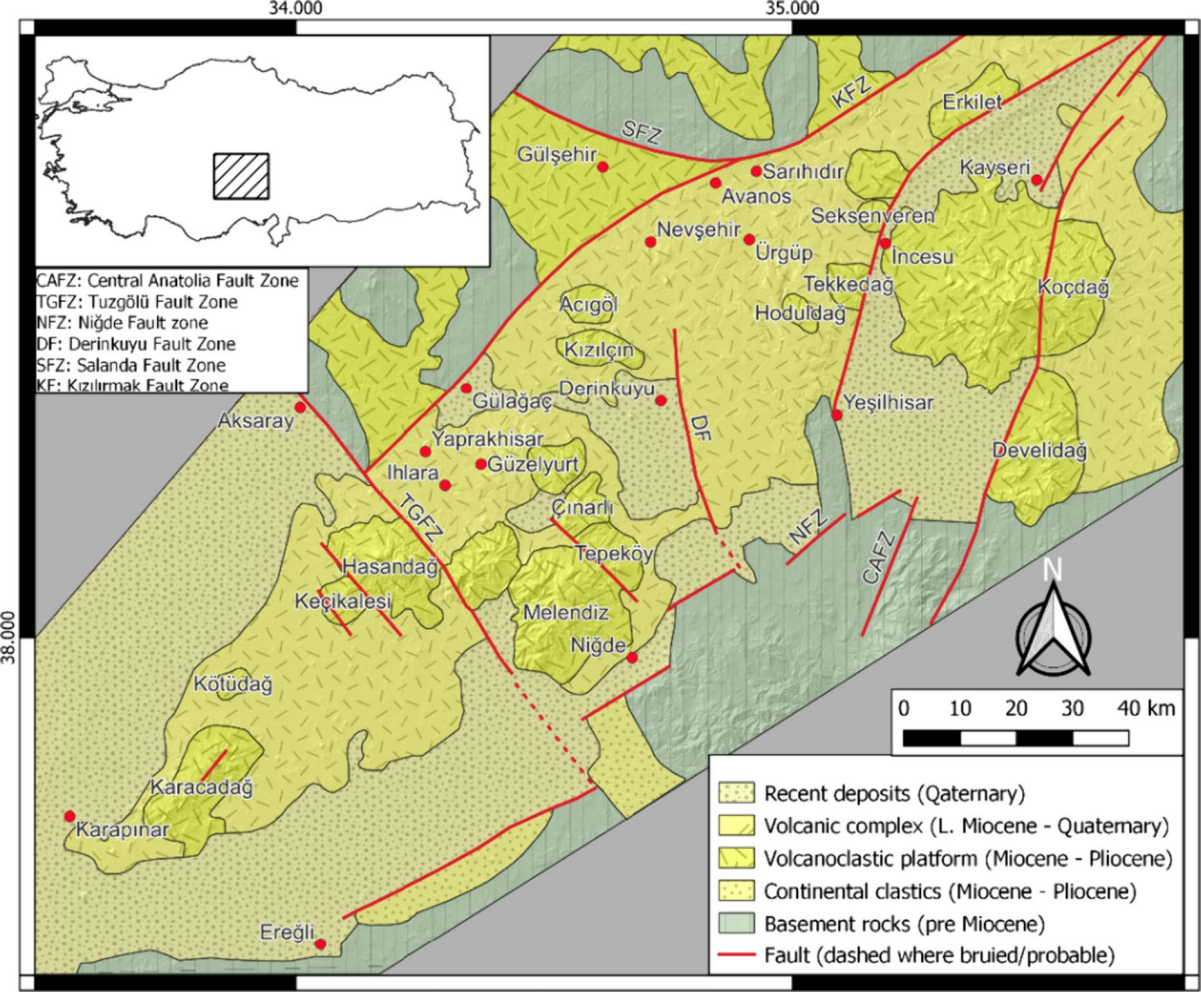
The study area's simplified geological map (Toprak, 2000)

The study's focus, Yaprakhisar and Balkayas travertines, are found in the CAVP (Toprak & Göncüoǧlu, 1993), which is a place where strike-slip and extensional component faults are active (Fig. 1). CAVP is located in the Central Anatolia region of Turkey and is a Neogene-Quaternary calc-alkaline volcanic region (Fig. 1). It has a long axis of almost 300 km. It runs as a belt from north to south (Toprak & Göncüoǧlu, 1993). Pre-Miocene basement rocks and Miocene-Quaternary volcanics of CAVP are two sets of geological units exposed in the research region. Both groups are covered by present-day continental deposits (Fig. 1) (Toprak & Göncüoǧlu, 1993).

### Pre-Miocene basement rocks

The Niğde Massif and Kırşehir Massif, from which the basement rocks originate, are located in the southeast and northwest. The Upper Cretaceous ophiolites are overlain by Paleozoic–Mesozoic moderate to high-grade metamorphic rocks, which cut through Upper Cretaceous-Paleocene plutonic rocks. In the research region, the massifs' cover unit is made up entirely of Eocene limestones, which rarely outcrop. The Tuzgolü and Central Anatolian fault zones are where the two narrow bands of Oligo-Miocene continental clastics are found (Toprak & Göncüoǧlu, 1993).

### Miocene-quaternary volcanics and sediments

Most of the area is covered by Miocene-Quaternary volcanics resulting from CAVP. They comprise nine volcanic complexes and a single volcaniclastic platform (Göncüoğlu & Toprak, 1992). The Late Miocene to Pliocene pyroclastic and epiclastic rocks make up the volcanoclastic platform, which is composed of continental (stream to lake) sediments interbedded with these rocks (Beekman, 1966; Innocenti et al., 1975; Pasquare, 1968) and exhibits substantial lateral and vertical facies variations (Toprak & Göncüoǧlu, 1993). Additionally, travertine formations from the Pleistocene to the Holocene age developed near faults in various parts of the CAVP.

### Sampling and processing

Nine samples were collected, six from the travertines in Balkayası and three from the travertines in Yaprakhisar. Nine samples from the local terrains were combined and processed to a mesh size of 100 in the laboratory at Yozgat Bozok University before being divided for sample separation and geochemical analysis. The PANalytical—Empyrean Multi-Purpose X-ray Diffractometer (MP-XRD) at the Yozgat Bozok University Science and Technology Application and Research Center (BILTEM) was used to examine the identical materials. XRD studies were carried out under Cu tube, 45 kV, 40 mA, 1.54,060 A0 (CuKa1) wavelength, and 37,9950 scan step rate conditions. At the Bureau Veritas [Automaticity in Cognition Motivation & Evaluation (ACME)] (Canada) Laboratory, geochemical analyses were carried out. Inductively Coupled Plasma-Mass Spectrometer (ICP-MS) and Inductively Coupled Plasma-Emission Spectrometer (ICP-ES) were used to examine the significant oxide groups and the total trace elements.

### Consumption does to the human

It is essential to evaluate exposure to radioactive material absorbed by individuals. Therefore, some parameters could be defined to understand the potential effects of natural radioactive materials. Usually, 226Ra activity (ARa), 232Th activity (ATh), and ^40^K activity (A_K_) could be used to get radiological parameters. Absorbed gamma dose rate (D), which indicates the radiation dose per hour at the height of 1 m above the ground, was defined as the following equation (Abdul Adziz & Khoo, 2018; Bilgici et al., 2022);

1 $$D=0.462{A}_{Ra}+0.604{A}_{Th}+0.042{A}_{K}$$

where the units of D in nGy/h and the activities of ^226^Ra, ^232^Th, and ^40^K were in Bq/kg. The minimum detection limits (MDL) values for D were referenced from the results of the Bilgici Cengiz & Caglar (2022).

In addition, radium equivalent activity (Ra_eq_) was used for the assessment of radiological exposure of radioactivity in geological materials could be defined by using following equation (Júnior et al., 2021; Nguyen & Trinh, 2022; Tufail, 2012);

2 $${Ra}_{eq}= {A}_{Ra}+1.43{A}_{Th}+0.077{A}_{K}$$

The minimum detection limits (MDL) values for Ra_eq_ were referenced from the results obtained from the Nguyen & Trinh (2022). Some parts of human body such as gonads, bone marrow and bone cells could be affected more from the radioactive materials than the other parts of the body. Therefore, the annual gonadal dose equivalent (AGDE) was defined as following formula (Penabei et al., 2018; Sivakumar et al., 2018);

3 $$AGDE=3.09{A}_{Ra}+4.18{A}_{Th}+0.314{A}_{K}$$

The minimum detection limits (MDL) values for AGDE were referenced from the results obtained from the Sivakumar (2018). Exposure rate (ER) was defined as the measure of ionizations in air produced by gamma rays per hour (Marsac et al., 2016). The exposure rate (ER) of a geological sample can be calculated by using the following equation (Onjefu et al., 2021);

4 $$ER=1.90{A}_{Ra}+2.82{A}_{Th}+0.179{A}_{K}$$

The minimum detection limits (MDL) values for ER were referenced from the results obtained from the Onjefu et al. (2021). Total Annual Effective Dose Equivalent (total) represents the annual absorbed dose by a person who spent time about 20% indoor (AEDEindoor) and 80% outdoor (AEDEoutdoor) in a year. AEDE_total_, AEDE_indoor_ and AEDE_outdoor_ values can be calculated by following equations (Avwiri et al. 2014; Suresh et al., 2022);

5 $$ AEDE_{indoor} = D \times 8760\;{\text{h}} \times 0.8 \times 0.7 \;{\text{SvGy}}^{ - 1} \times 10^{ - 6} $$

6 $$ AEDE_{outdoor} = D \times 8760\;{\text{h}} \times 0.2 \times 0.7\;{\text{SvGy}}^{ - 1} \times 10^{ - 6} $$

7 $${AEDE}_{total}={AEDE}_{indoor}+{AEDE}_{outdoor}$$

The minimum detection limits (MDL) values for AEDE were referenced from the results obtained from the Suresh et al. (2022).

Excess lifetime cancer risk (ELCR) represents the additional probability of anticipated cancer cases within the public due to their exposure to a radiation dose. ELCR can be determined by following the formula (Avwiri et al. 2014; Awad et al., 2022);

8 $$ ELCR = AEDE_{total} \times 70 \times 0.05 $$

The minimum detection limits (MDL) values for ELCR were referenced from the results obtained from the Awad et al. (2022).

The internal hazard index (H_in_) and the external hazard index (H_ex_) were defined by Beretka and Mathew in 1985 by following equations (Beretka & Mathew, 1985; Sandu et al., 2020);

9 $${H}_{in}= \frac{{A}_{Ra}}{185}+\frac{{A}_{Th}}{259}+\frac{{A}_{K}}{4810}$$

10 $${H}_{ex}= \frac{{A}_{Ra}}{370}+\frac{{A}_{Th}}{259}+\frac{{A}_{K}}{4810}$$

Gamma representative level (GRL) refers to assessment on the potential risk and level of hazard associated with exposure to gamma radiation. GRL can be calculated by using the following formula (Ibraheem et al., 2018);

11 $$GRL= \frac{{A}_{Ra}}{150}+\frac{{A}_{Th}}{100}+\frac{{A}_{K}}{1500}$$

The minimum detection limits (MDL) values for H and GRL were referenced from the results obtained from the Sandu et al. (2020).

### Statistical analysis (Kriging)

The kriging technique is an interpolation method extensively applied in geostatistics. Employing regional variables, this approach estimates individual points and broader spatial contexts. Its central objective is to mitigate estimation discrepancies through variance minimization, contributing to the method's widespread popularity. Moreover, the distinguishing factors of the Kriging approach encompass the interdependence of variables and observations grounded in the premise of random sampling, prominently elevating its significance in geostatistical predictions. The Kriging method frequently applies, particularly in geographical data analysis and geological investigations. It offers an effective strategy to complete missing or sparse data by leveraging spatial associations within datasets. This enables the calculation of predictive values for specific locations while minimizing forecasting errors. The formulation represented as Eq. (12) serves to predict new points using the Kriging method. This equation operates by incorporating spatial relationships and associated weights among distinct observations. Here, x_0_ denotes a novel prediction point, with f(x_0_) signifying the resultant predictive value. The parameter 'n' signifies the total samples, and w_i_(x_0_) represents the pertinent weights. Using this equation, one can calculate the projected value for a fresh location (Bilici et al., 2020; Külahcı & Bilici, 2019; Oliver & Webster, 2015).

12 $$f\left({x}_{0}\right)=\sum_{i=1}^{n}{w}_{i}\left({x}_{0}\right)f\left({x}_{i}\right)$$

Illustratively employed in this study, the Kriging technique facilitated the creation of surface maps delineating the radioactivity of ^232^Th, ^40^K, and ^137^Cs (Granek, 2011). This application holds pivotal import, aiding in comprehending and cartographically representing the dispersion of radioactive materials within geological or environmental analyses. For instance, estimating the dispersion of such radioactive elements within soil or water and discerning inter-regional disparities finds substantial support from the Kriging approach. Consequently, the Kriging method emerges as a frequently employed instrument within geostatistical analysis and spatial estimation endeavors (Bilici et al., 2020). It seeks to curtail the discrepancies intrinsic to estimations by effectively integrating variable relationships. Thus, it finds recurrent favor in realms such as geographical data analysis, geology, environmental sciences, and wherever stochastic sampling assumptions and spatial interdependencies come into play.

## Results and discussion

### Mineralogy

**Fig. 2:**
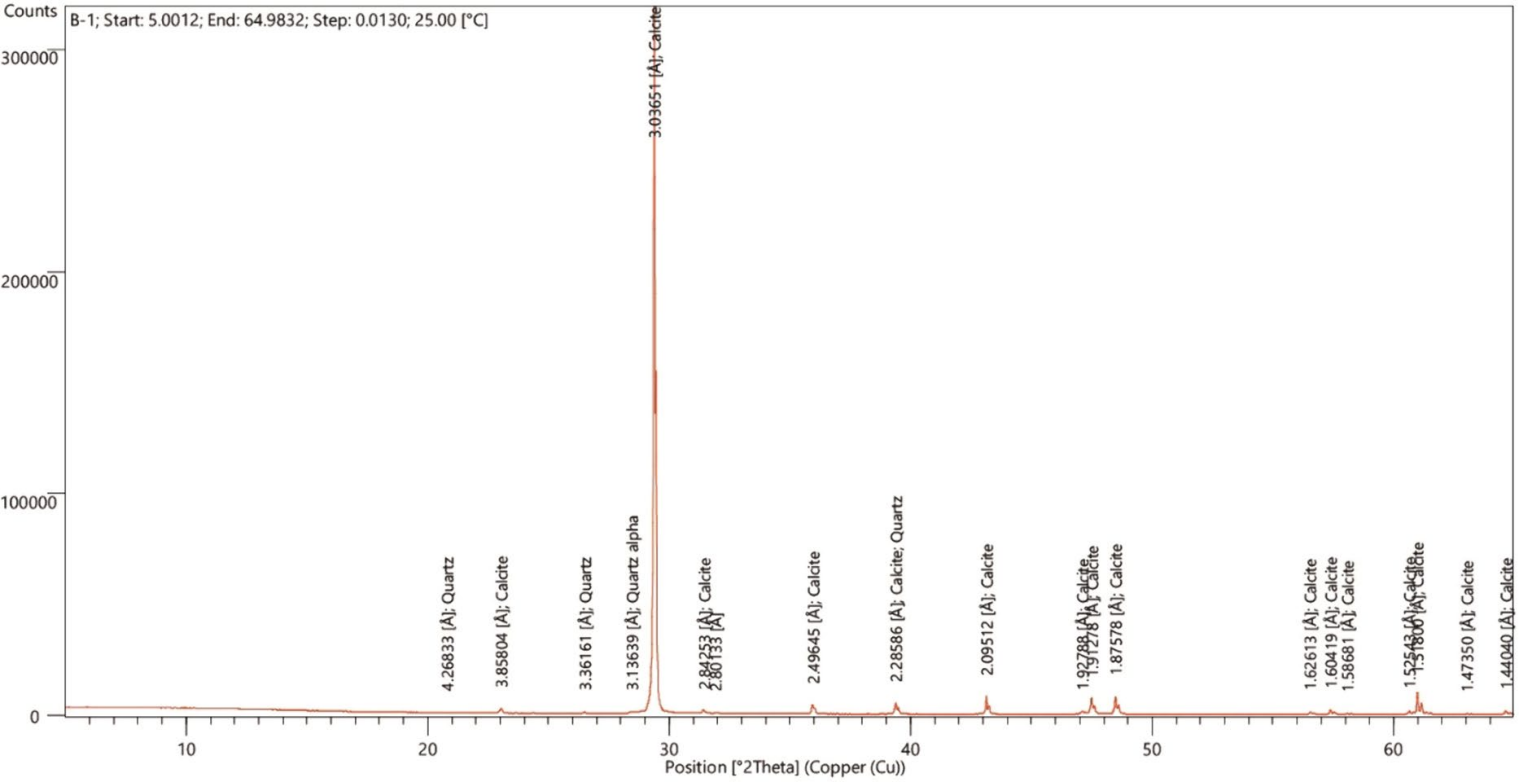
The B-1 sample's XRD diagram

Almost all travertines comprise two primary polymorphs with aragonite and calcite chemical compositions. According to XRD analyses of the samples from the study region, calcite (CaCO_3_) and trace amounts of quartz (SiO_2_) minerals were found (Fig. 2).

### Chemical composition

In this study, the trace element ratios in travertines collected from the Yaprakhisar region with codes B1-M, B2, B5, B7, B8, and B10, as well as from Balkayasi with codes T5-M, T12, and Z1, have been provided in Table 1 in parts per million (ppm) and as percentages. The results of the chemical analysis performed at Bureau Veritas Mineral Laboratories Acme Analytical Lab (Canada) are given in Table 1. The CaO ratio displayed high values aligned with the chemical composition and mineralogy.

**Table 1:** Major and trace element content of Yaprakhisar and Balkayası travertines

Sample	B-1M*	B-2*	B-5*	B-7*	B-8*	B-10*	T5M**	T12**	Z1**
SiO_2_ (%)	0.16	0.04	0.22	0.16	0.06	0.97	0.52	0.01	< 0.01
Al_2_O_3_ (%)	0.01	0.01	0.01	0.02	0.01	0.25	0.16	< 0.01	< 0.01
Fe_2_O_3_ (%)	0.04	0.04	0.04	0.04	0.04	0.1	0.9	0.88	0.17
MgO (%)	0.15	0.13	0.16	0.14	0.16	0.26	0.37	0.49	0.58
CaO (%)	55.53	55.6	55.44	55.59	56.01	54.81	54.26	54.83	55.19
Na_2_O (%)	0.01	0.01	0.01	0.01	0.01	0.02	0.06	0.06	0.04
K_2_O (%)	0.01	0.01	0.01	0.01	0.01	0.04	0.03	< 0.01	< 0.01
TiO_2_ (%)	0.01	0.01	0.01	0.01	0.01	0.01	< 0.01	< 0.01	< 0.01
P_2_O_5_ (%)	0.02	0.02	0.02	0.02	0.01	0.01	0.03	0.01	< 0.01
MnO (%)	0.32	0.35	0.2	0.3	0.03	0.12	0.07	0.08	< 0.01
Cr_2_O_3_ (%)	0.002	0.002	0.002	0.002	0.002	0.002	< 0.002	< 0.002	< 0.002
Ba (ppm)	275	364	262	166	120	1793	48	17	30
Sr (ppm)	303.7	365.2	361.5	239.2	229.3	350.9	935.7	740.6	655.7
Sc (ppm)	2	5	2	2	1	1	3	< 1	< 1
Be (ppm)	1	8	1	1	1	1	5	7	< 1
Co (ppm)	0.7	1.7	1.2	1.2	1.6	1.1	3.6	1.8	3
Cs (ppm)	0.1	0.1	0.1	0.1	0.1	1.7	5.3	< 0.1	< 0.1
Nb (ppm)	0.2	0.2	0.2	0.1	0.3	0.2	0.2	< 0.1	0.2
Rb (ppm)	0.1	0.1	0.1	0.1	0.1	2.4	4.3	< 0.1	< 0.1
Th (ppm)	0.2	0.2	0.2	0.2	0.2	0.2	n.d	n.d	n.d
U (ppm)	0.1	0.1	0.1	0.1	0.1	0.1	n.d	n.d	n.d
LOI	43.7	43.8	43.9	43.7	43.7	43.2	43.5	43.5	43.9

*Temiz et al. (2021), **Temiz et al. (2018)

### Radiation hazard parameters

The results of this study yielded minimum detectable activities (MDAs): Bq/kg for 0.508 Bq/kg for ^232^Th, 3.078 Bq/kg for ^40^K, and 0.044 Bq/kg for ^137^Cs. The activity concentrations of ^232^Th, ^40^K, and ^137^Cs were calculated and compared with the average values from this study and the global average values, as shown in Table 2.

**Table 2:** Activity concentration of ^232^Th, ^40^K and ^137^Cs (Bq/kg)

	Sample No	^232^Th	^40^K	^137^Cs
Yaprakhisar stations	B1-M	2.09 ± 0.28	8.10 ± 0.94	0.48 ± 0.07
	B2	3.62 ± 0.37	7.90 ± 0.92	0.42 ± 0.06
	B5	12.07 ± 0.67	n.d	2.54 ± 0.15
	B7	5.63 ± 0.39	4.21 ± 0.57	1.44 ± 0.10
	B8	7.55 ± 0.52	2.88 ± 0.54	2.09 ± 0.13
	B10	3.26 ± 0.31	10.83 ± 0.95	1.66 ± 0.11
Balkayasi stations	T5-M	9.23 ± 0.51	4.22 ± 0.59	3.26 ± 0.15
	T12	6.55 ± 0.44	5.62 ± 0.70	1.72 ± 0.11
	Z1	6.18 ± 0.40	13.41 ± 1.00	2.52 ± 0.12
	Average value of this study	6.21 ± 0.43	7.15 ± 0.78	1.79 ± 0.11
	World average value [1]	45	412	–

The radioactivity levels in waste samples were measured using a high-purity germanium (HpGe) detector cooled with liquid nitrogen, located in the Department of Physics at Akdeniz University (Eke et al., 2019). The spectra were collected and analyzed using an MC^2^ analyzer and software (MC^2^ Analyzer, 2022). The background spectrum was counted over a day and subtracted from each sample spectrum to eliminate background effects and obtain accurate results.

**Fig. 3:**
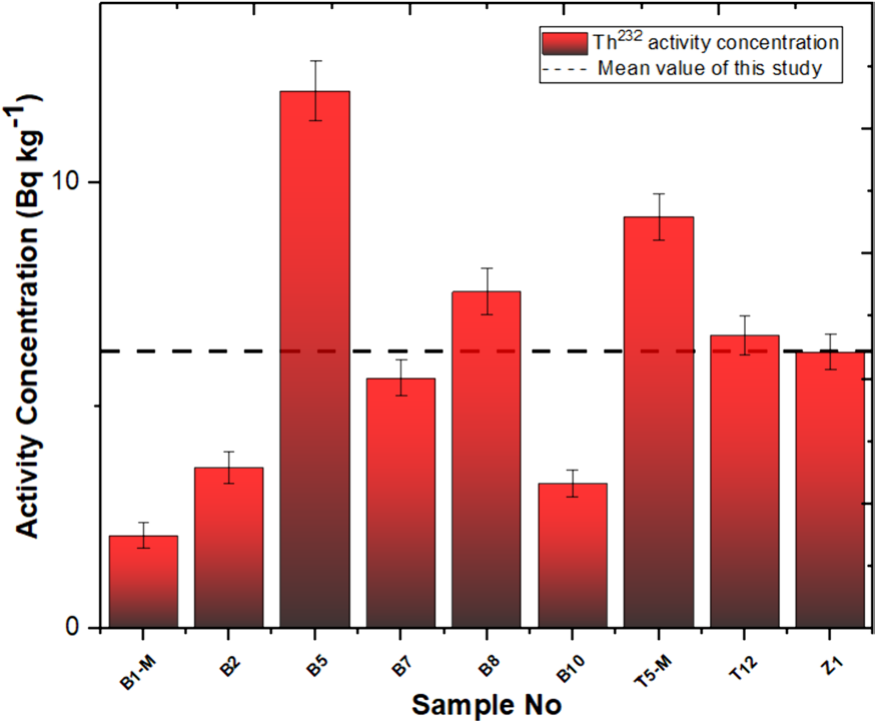
^232^Th activity concentration, the mean value of this study (Shayeb & Baloch, 2020)

**Fig. 4:**
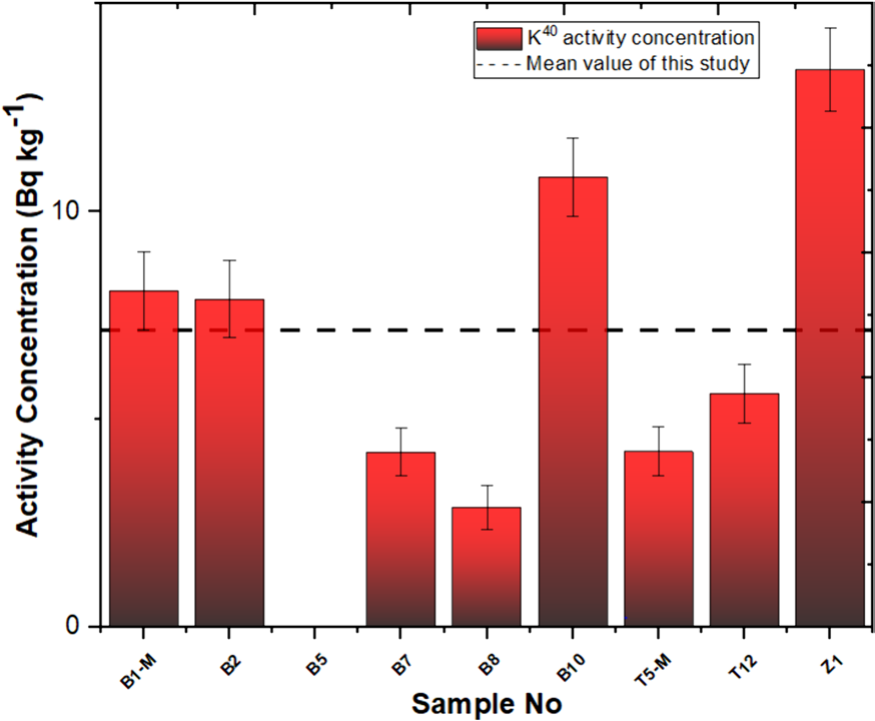
^40^K activity concentration, mean value of this study

**Fig. 5:**
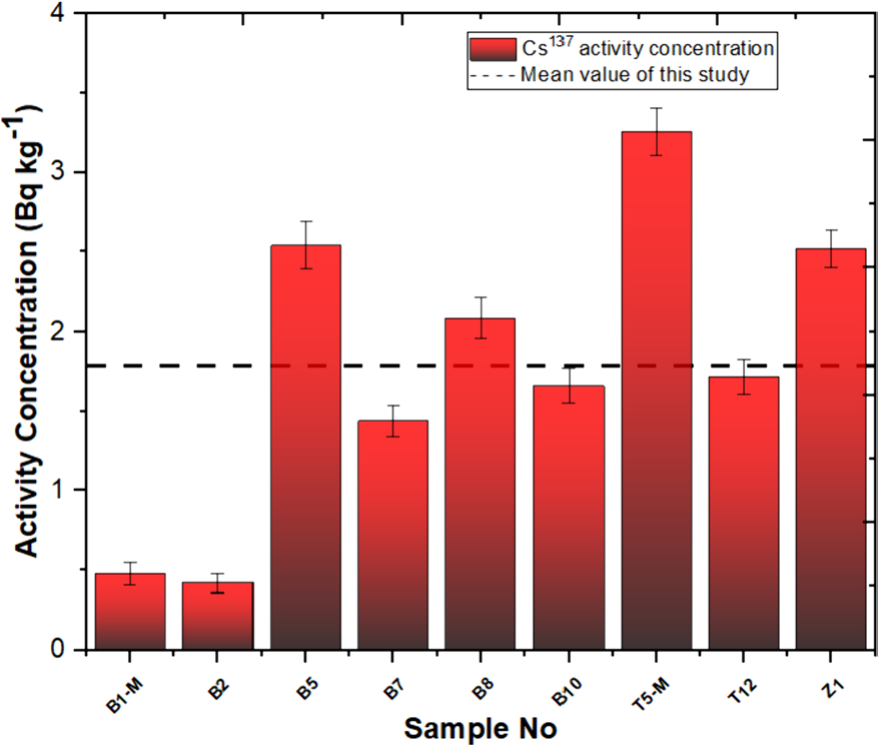
^137^Cs activity concentration, mean value of this study

In this investigation, the measurement of natural and artificial radionuclide activities obtained for travertines is given in Table 2. Based on the findings derived from our investigation, the Yaprakhisar region exhibited the highest concentration of ^232^Th at the B5 station, whereas the lowest ^232^Th activity concentration was observed at the B1-M station. Similarly, in the Balkayasi region, the T5-M station recorded the highest ^232^Th activity concentration, while the Z1 station exhibited the lowest ^232^Th concentration. The UNSEAR provides a global mean of 45 Bq/kg for ^232^Th. As depicted in Fig. 3, the concentrations of ^232^Th in the travertine samples collected from nine stations in this research fall below this designated threshold (UNSCEAR, 2008). Similarly, when examining the ^40^K activity concentration levels, the Yaprakhisar region displayed the highest ^40^K activity concentration at the B10 station. In contrast, the lowest ^40^K activity concentration was observed at the B8 station. Furthermore, no detectable levels of 40K activity concentration were recorded at station B5. In the Balkayasi region, the Z1 station recorded the highest ^40^K activity concentration, while the T5-M station exhibited the lowest ^40^K concentration. The UNSEAR has established a global average of 412 Bq/kg for ^40^K activity concentration. As illustrated in Fig. 4, the ^40^K activity concentrations in the travertine samples collected from nine stations during this study are below this specified threshold (UNSCEAR, 2008). At the Yaprakhisar station, B5 registers the highest level of ^137^Cs activity concentration. In contrast, station B2 exhibits the lowest 137Cs activity concentration, as indicated in Fig. 5. Meanwhile, at the Balkayasi station, T5-M shows the highest 137Cs activity concentration level. In contrast, station T12 displays the lowest ^137^Cs activity concentration, as illustrated in Fig. 5.

**Fig. 6:**
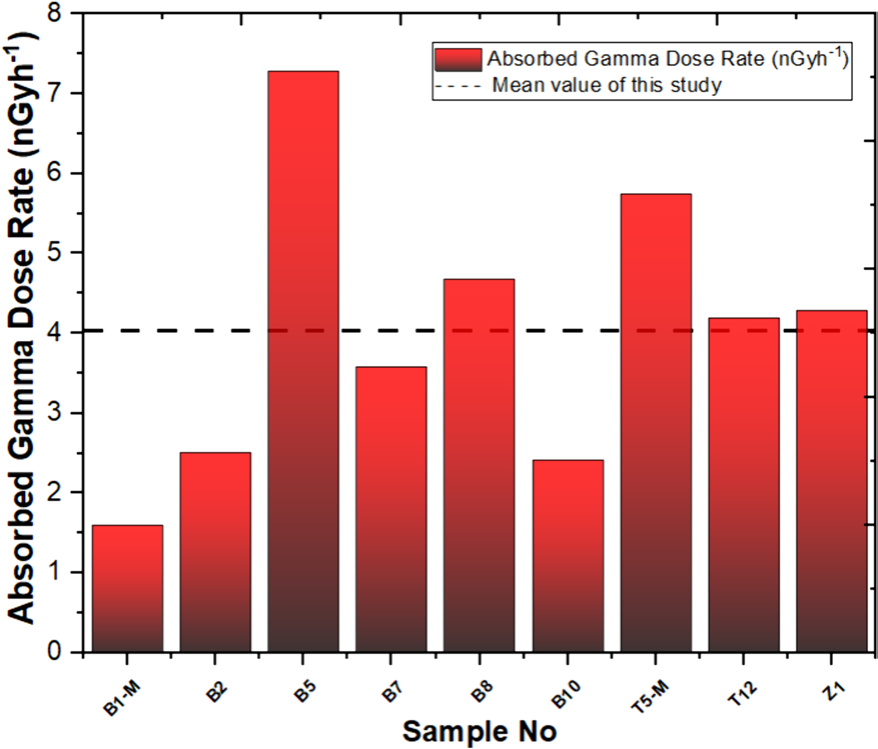
Absorbed gamma dose rate and mean value of this study

The calculated values for D, Ra_eq_, AGDE, ER, AEDE_total_, GRL, H_in,_ and H_ex_ for the stations in this study are presented in Table 3. The mean absorbed gamma dose rate (D) value was calculated as 4.03 nGy/h, whereas the UNSEAR report states an average D value of 59 nGy/h. It can be observed that the calculated D value is lower than the one provided in the UNSEAR report for both stations, Yaprakhisar and Balkayasi. Based on the findings derived from our investigation, the Yaprakhisar region exhibited the highest value of D at the B5 station. In contrast, the lowest D absorbed dose value was observed at the B1-M station. Similarly, in the Balkayasi region, the T5-M station recorded the highest D absorbed dose value, while the T12 station exhibited the lowest D value, as shown in Fig. 6.

**Table 3:** D, Ra_eq_, AGDE, ER, AEDE_total_, GRL, H_in,_ and H_ex_ of studied samples

Sample Code	D (nGy/h)	Ra_eq_ (Bq/kg)	AGDE (µSv/y)	ER (µR/h)	AEDE_total_ (mSv/y)	ELCR_total_ (× 10^–3^)	GRL	H_in_	H_ex_
B1-M	1.60	3.61	11.29	7.35	0.010	0.034	0.03	0.01	0.01
B2	2.51	5.78	17.60	11.62	0.015	0.054	0.04	0.02	0.02
B5	7.29	17.26	50.44	34.03	0.045	0.156	0.12	0.05	0.05
B7	3.58	8.38	24.87	16.64	0.022	0.077	0.06	0.02	0.02
B8	4.68	11.01	32.45	21.80	0.029	0.100	0.08	0.03	0.03
B10	2.42	5.50	0.00	0.00	0.015	0.052	0.00	0.00	0.01
T5-M	5.75	13.52	39.89	26.77	0.035	0.123	0.10	0.04	0.04
T12	4.19	9.80	29.14	19.48	0.026	0.090	0.07	0.03	0.03
Z1	4.29	9.87	30.03	19.82	0.026	0.092	0.07	0.03	0.03
Mean value	4.03	9.41	26.19	17.50	0.025	0.087	0.06	0.02	0.03

**Fig. 7:**
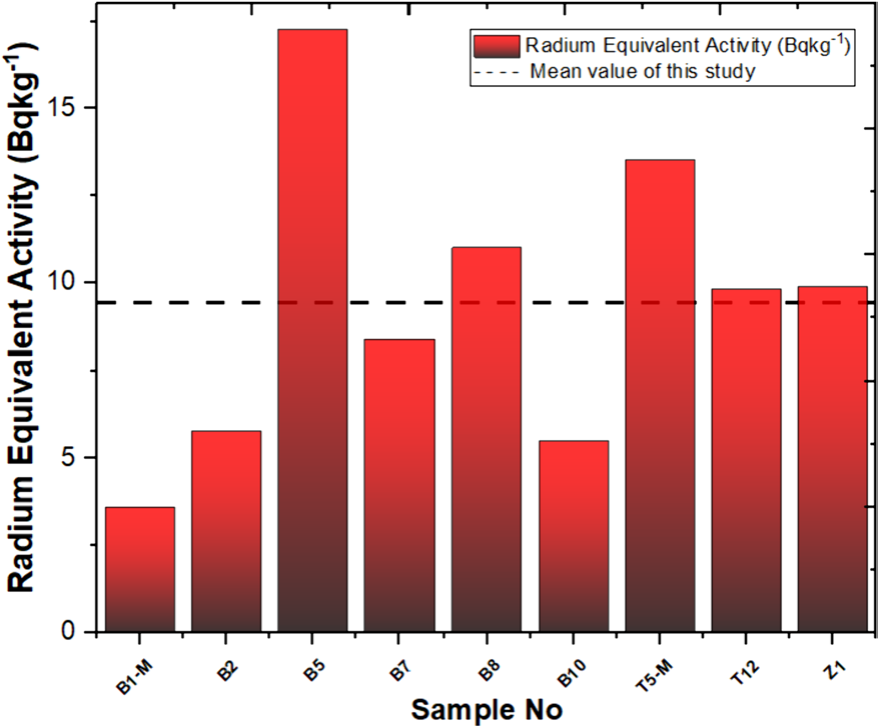
Radium equivalent activity and mean value of this study

Calculated values of Ra equivalent activities for radionuclide activity concentrations are given in Fig. 7 for nine stations. The mean radium equivalent activity (Ra_eq_) value was computed as 9.41 Bq/kg, lower than the Ra_eq_ 370 Bq/kg value determined by NEAOECD (NEA, 1979). The calculated Ra equivalent activity values based on radionuclide activity concentrations are depicted in Fig. 7 for nine stations. In the Yaprakhisarı region, station B5 exhibits the highest Ra_eq_ value, while station B1-M shows the lowest value. It is worth noting that these values vary in tandem with the D values. As for the Balkayasi region, station T5-M records the highest Ra_eq_ value, while station T12 reports the lowest value, and similarly, these values exhibit a parallel relationship with the D values.

When the study analysis is compared with similar content in the literature, in studies conducted by Abbasi and colleagues, the analysis results of samples taken from the soil surface indicated that radiation hazard and natural radioactivity levels were higher than the global average (Abbasi, 2022; Abbasi et al., 2020a, 2020b, 2022a, 2022b, 2022c). Nevertheless, it has been determined that no risk is associated with its use as a building construction material. Moreover, considering the importance of the area from which this study was conducted, various natural and artificial radioactivity measurement studies have been performed in this region due to its proximity to critical structures, such as the Akkuyu Nuclear Power Plant in Turkey (Abbasi, 2023; Abbasi et al., 2023). Furthermore, in the study area selected in Turkey, which is surrounded by seas on three sides, the results of radiation risk assessments conducted in the nearest seas have also been closely monitored, and all these studies have been evaluated as safe since they do not exceed the global average (Abbasi et al., 2022a, 2022b, 2022c, 2020a, 2020b; Kefalati et al., 2021).

**Fig. 8:**
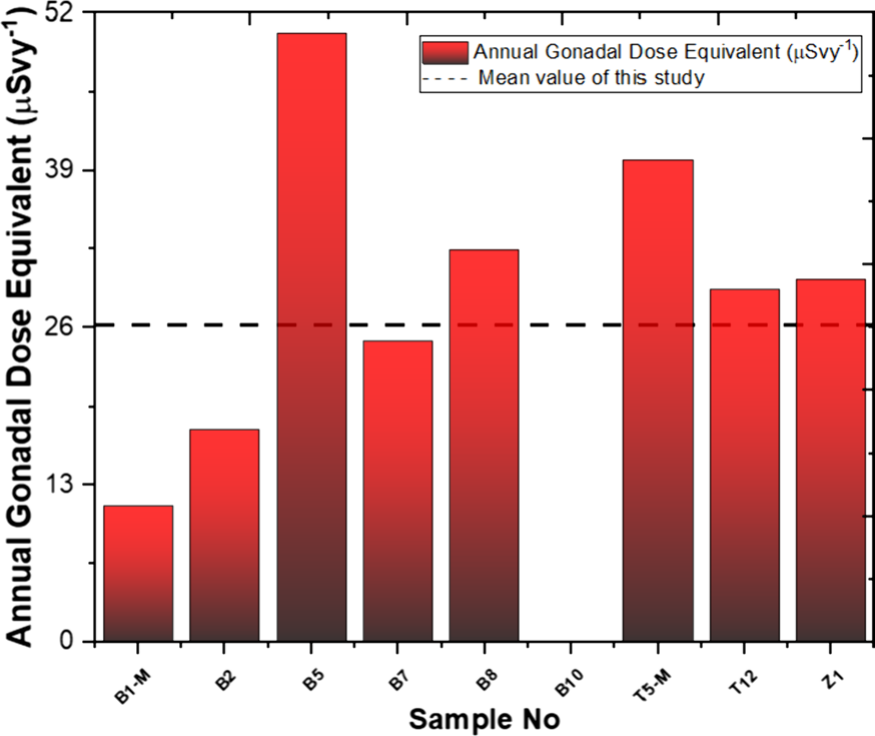
Annual gonadal dose equivalent and mean value of this study

The average AGDE values calculated for nine stations in this study amount to 26.19 µSv/y, indicating a value significantly lower than the global average of 300 µSv/y (Darwish et al., 2015; Senthilkumar & Narayanaswamy, 2016). In the Yaprakhisarı region, station B5 exhibits the highest AGDE value, while station B1-M shows the lowest value. Also, it was impossible to calculate the AGDE value for station B10. As for the Balkayasi region, station T5-M records the highest AGDE value, while station T12 reports the lowest value. The calculated values are displayed in Fig. 8.

**Fig. 9:**
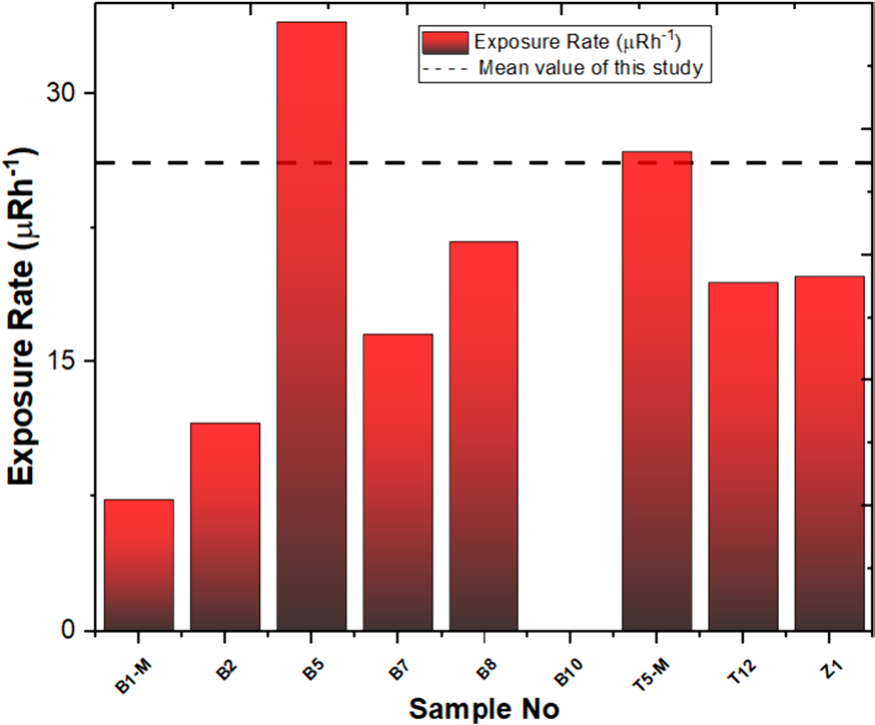
Exposure rates and mean value of this study

The average ER values calculated for nine stations in this study amount to 17.50 µR/h, indicating a value significantly lower than the global average of 600 µR/h (UNSCEAR, 2000). In the Yaprakhisarı region, station B5 exhibits the highest ER value, while station B1-M shows the lowest value. Also, it was impossible to calculate the ER value for station B10. As for the Balkayasi region, station T5-M records the highest ER value, while station T12 reports the lowest value. The calculated values are displayed in Fig. 9.

**Fig. 10:**
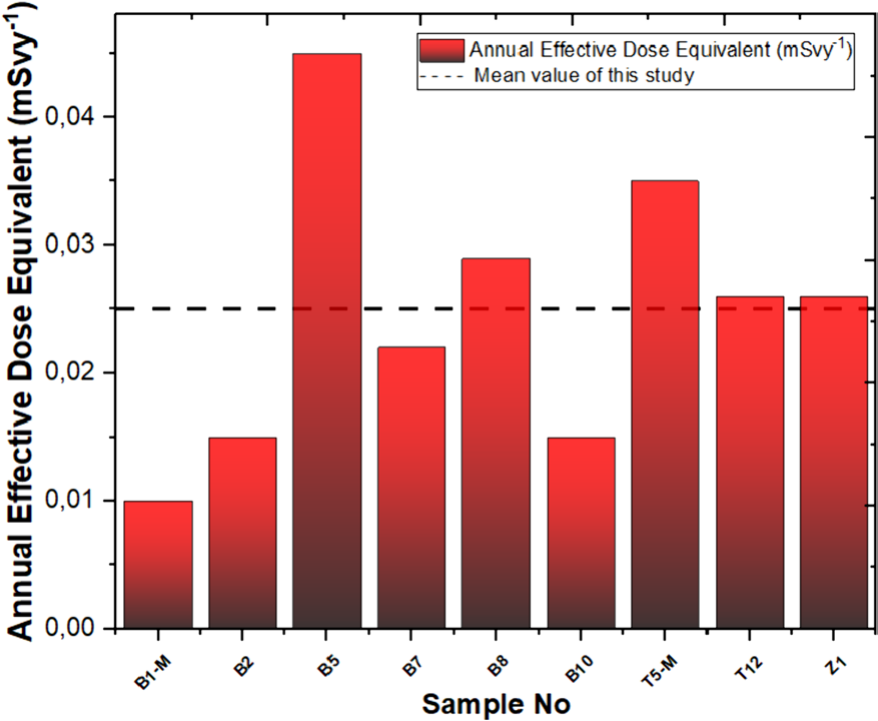
Annual effective dose equivalent and mean value of this study

The calculated AEDE dose equivalent values for nine stations in this study are shallow, averaging only 0.025 µSv/y. In Fig. 10, the lowest value is the global average of 7.47 µSv/y for these nine stations (UNSCEAR, 2000). In the Yaprakhisarı region, station B5 stands out with the highest AEDE value, while station B1-M exhibits the lowest among them. Moving to the Balkayasi region, stations T5-M record the highest AEDE value, whereas stations T12 and Z1 report the lowest values in this area.

**Fig. 11:**
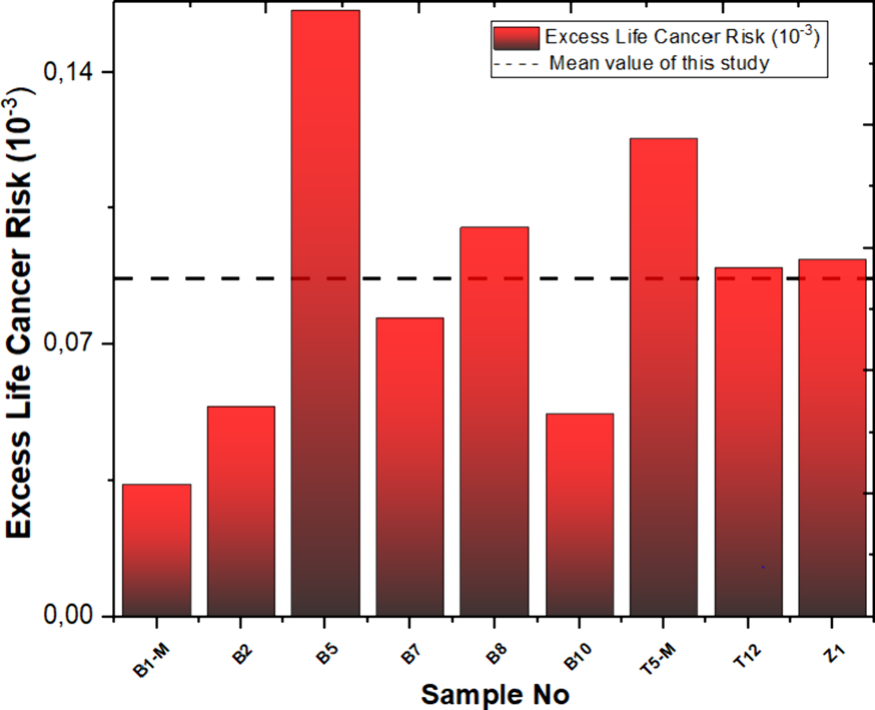
Excess life cancer risk and mean value of this study

The mean ELCRtotal of studied samples is 0.087 × 10^–3^, which is higher than the world average value of 0.29 × 10^–3^ (Abdullahi et al., 2019; Shayeb & Baloch, 2020). In the Yaprakhisarı region, station B5 stands out with the highest ELCRt_otal_ value, while station B1-M exhibits the lowest among them. Moving to the Balkayasi region, station T5-M records the highest ELCRt_otal_ value, whereas station T12 reports the lowest values in this area. The calculated values are displayed in Fig. 11.

**Fig. 12:**
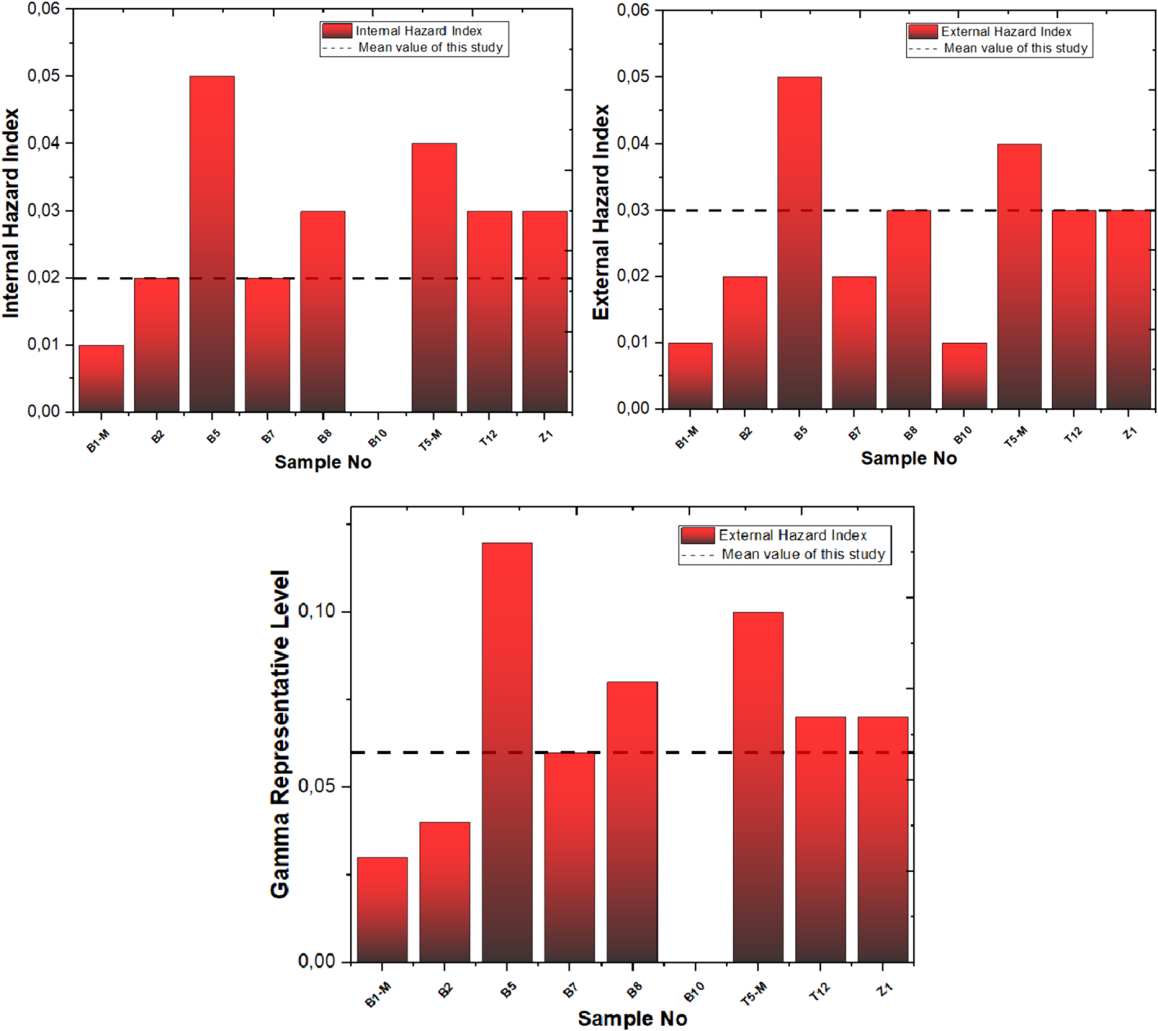
Hin, Hex, and GRL values of studied samples and permissible limit value

The mean values of H_in_, H_ex_, and GRL for the studied samples are as follows: 0.02, 0.03, and 0.06, respectively. These values are considerably lower than the permissible limit value of 1 (El-Tahawy & Higgy, 1995). The calculated values are displayed in Fig. 12. Within the Yaprakhisarı region, station B5 distinguishes itself with the highest H_in_ and H_ex_ values, while station B1-M displays the lowest values in comparison. Shifting our focus to the Balkayasi region, stations T5-M record the highest H_in_ and H_ex_ values, while stations T12 and Z1 report the lowest values. In the Yaprakhisarı region, station B5 stands out with the highest GRL value, while station B1-M exhibits the lowest GRL value in comparison. Additionally, station B10 has recorded a GRL value of 0. Shifting our attention to the Balkayasi region, stations T5-M record the highest GRL value, whereas stations T12 and Z1 report the lowest GRL values.

### Statistical analysis results

**Fig. 13:**
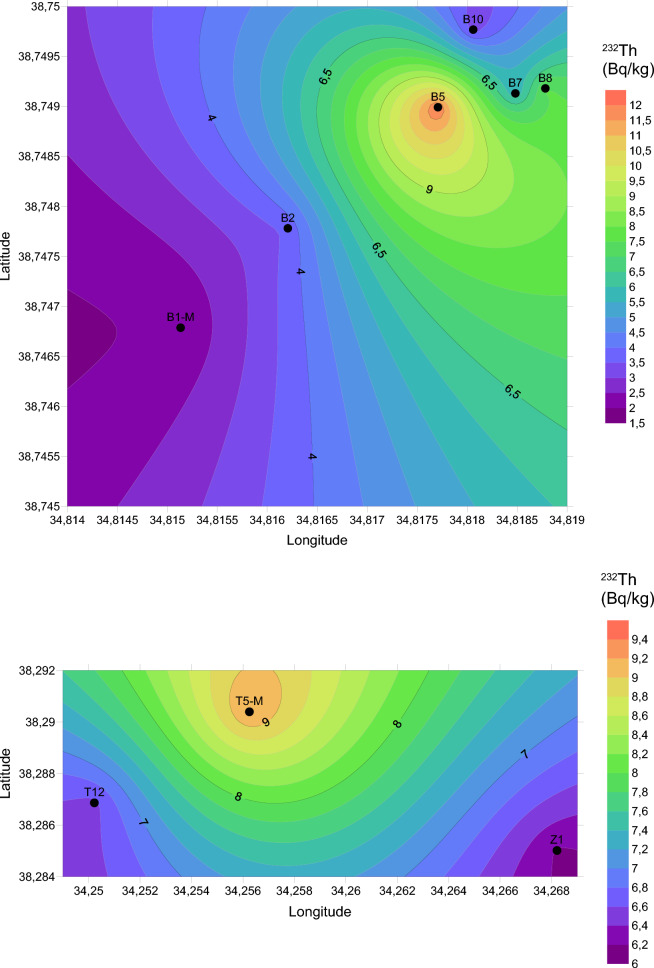
Spatial distribution map of ^232^Th in the travertines of Yaprakhisar and Balkayası

**Fig. 14:**
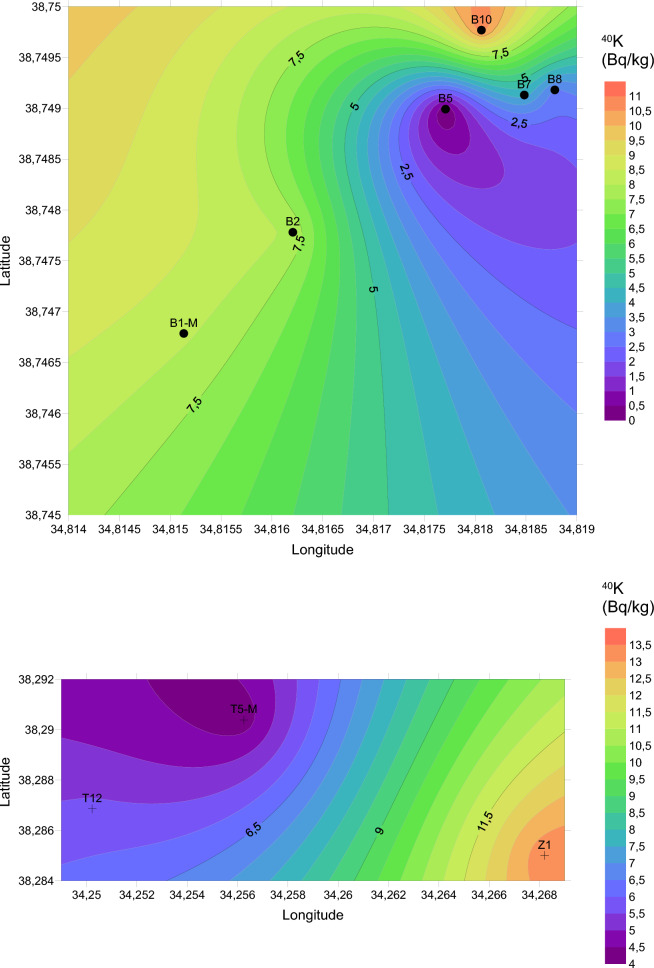
Spatial distribution map of ^40^K in the travertines of Yaprakhisar and Balkayası

**Fig. 15:**
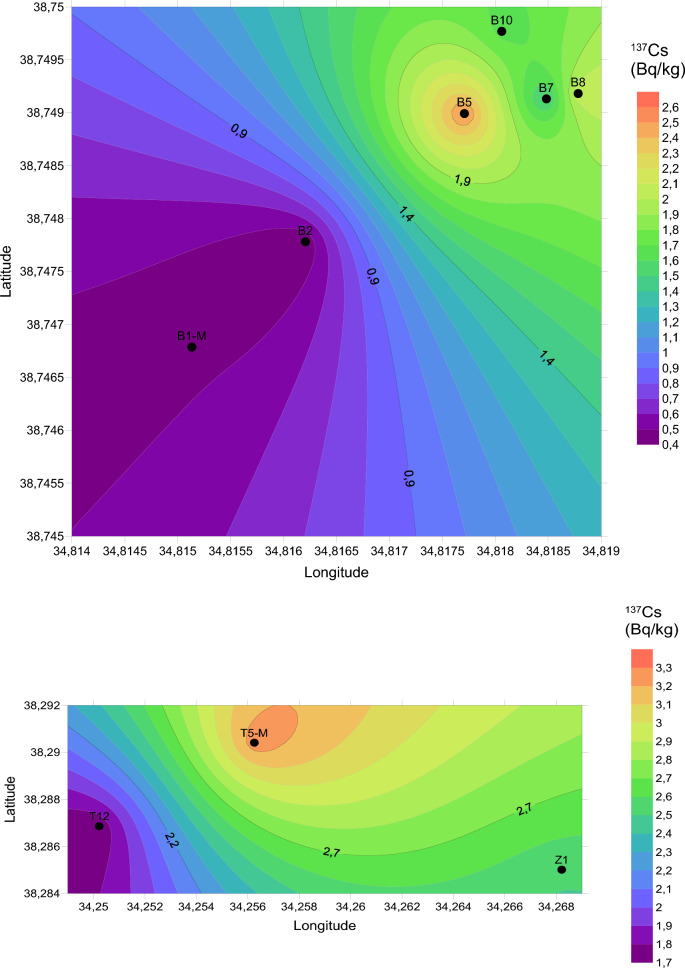
Spatial distribution map of ^137^Cs in the travertines of Yaprakhisar and Balkayası

In this study, the surface graphs of the ^232^Th, ^40^K, and ^137^Cs data measured for the transverters are given in Figs. 13, 14 and 15. The ^232^Th, ^40^K, and ^137^Cs activity concentrations shown in Table 2 were mapped with the help of Surfer using the Kriging method. Within these maps, the color scale has been meticulously designed to follow a gradient from red to purple, aligning with the progression observed in the visible light spectrum. It's worth highlighting that, to represent the data for each radionuclide accurately, distinct upper and lower limit values have been established for each map. The maximum and minimum values on the maps in Figs. 3, 4 and 5 are indicated in red and purple, respectively.

## Conclusions

In this research, we calculated the activity concentrations of ^232^Th, ^40^K, and ^137^Cs in the samples collected from the Yaprakhisa and Balkayasi travertines in the Cappadocia region of Turkey using an HPGe detector. Furthermore, the study examined the potential environmental impact of radiation emanating from these isotopes (^232^Th, ^40^K, and ^137^Cs) on the health of the local population by assessing various radiological impact parameters, including D, Ra_eq_, AGDE, ER, AEDE_total_, ELCR, H_in_, H_ex_, and GRL. The calculated average radioactivity levels are 6.21 ± 0.43 Bq/kg for ^232^Th and 7.15 ± 0.78 Bq/kg for ^40^K, while the count of artificial radionuclide is 1.78 ± 0.11 Bq/kg for ^137^Cs.

The study yielded several noteworthy findings, as outlined below:

i. In the investigation, it was observed that the B5 station in the Yaprakhisarı region exhibited the highest activity measurement. In contrast, the B1-M station had the lowest activity measurement, consistent with global average values. In the Balkayasi region, the station with the highest activity measurement was T5-M and the lowest was T12. Importantly, it was found that none of the stations exceeded the established limits in any instance.
ii. ^226^Ra was undetectable at all stations for this study area.
iii. Through a scientific comparison of the findings with global average values, it was observed that the results from this study remained within the specified limit values.
iv. The mean values for D, Ra_eq_, and AEDE_total_ were determined as 4.03 nGy/h, 9.41 Bq/kg, and 0.0251 µSv/y, respectively. Additionally, the mean values for Hex, Hin, and GRL were found to be 0.03, 0.02, and 0.06, respectively. Consequently, the average activity levels of 232Th and 40K in this study fell outside the range of global average values documented in scientific literature.

Consequently, upon evaluating the activity results from Yaprakhisar and Balkayasi, prominent travertine regions within Cappadocia that draw the attention of tourists, it becomes evident that the majority of the studied travertines do not present any health risks in comparison to the values documented in scientific literature. The assessment of all radiological parameters affirms the safety of these world-renowned travertines.
